# Mitochondrial Quality Control Systems in Septic AKI: Molecular Mechanisms and Therapeutic Implications

**DOI:** 10.7150/ijms.107012

**Published:** 2025-03-19

**Authors:** Ying Tan, Yue Ouyang, Zisheng Ma, Jianming Huang, Chuhong Tan, Junxiong Qiu, Feng Wu

**Affiliations:** 1 Department of Critical Care Medicine, Nanfang Hospital, Southern Medical University, Guangzhou 510515, China.; 2 Department of Critical Care Medicine, The First School of Clinical Medicine, Southern Medical University, Guangzhou 510515, China; 3 Department of Cardiovascular Surgery, Chinese PLA General Hospital, Beijing 100853, China.

**Keywords:** SGLT2i, LPS-mediated kidney damage, mitochondria, AMP.

## Abstract

**Objectives:** Despite significant advancements in medical treatments, the creation of a successful treatment strategy for acute kidney injury (AKI) remains a pressing concern. Given the well-documented clinical benefits of canagliflozin in renal protection, our research focused on exploring the possible therapeutic benefits of canagliflozin in treating AKI, with a focus on its underlying mechanisms of action.

**Methods:** To induce AKI, we utilized lipopolysaccharide (LPS) in the presence of canagliflozin, allowing us to assess the drug's effects on kidney function and structure.

**Results:** Our results indicate that canagliflozin lowered blood urea nitrogen and serum creatinine concentrations while enhancing tubular architecture in rodents with LPS-triggered septic AKI. It additionally diminished inflammation, oxidative damage, and tubular cell apoptosis. *In vitro*, canagliflozin maintained mitochondrial functionality in LPS-exposed HK-2 cells by stabilizing membrane potential, reducing ROS generation, and normalizing respiratory chain activity. Its benefits were facilitated through the AMPKα1/PGC1α/NRF1 axis, promoting mitochondrial regeneration. Importantly, blocking this pathway or employing AMPKα1-deficient animals negated canagliflozin's protective effects, highlighting the essential role of AMPKα1 in its kidney-protective mechanisms.

**Conclusion:** Our investigation implies that canagliflozin might represent a viable treatment strategy for septic AKI, operating through the stimulation of the AMPKα1/PGC1α/NRF1 axis to preserve kidney performance and structural integrity. These findings warrant further investigation into the clinical potential of canagliflozin in this context.

## Introduction

AKI's clinical manifestations vary depending on the underlying reasons while this condition is typically characterized by three primary features 1. The AKI's occurance is significant, affecting approximately 14% of hospitalized patients, with a substantially higher prevalence of 50% observed in individuals with pre-existing renal dysfunction 2. Consequently, there exists an urgent necessity to investigate innovative treatment options which may effectively treat LPS-caused renal dysfunction, with the ultimate goal of reducing mortality rates among affected patients.

Canagliflozin is a novel SGLT2 inhibitor that reduces glucose accumulation through elevating urinary glucose release 3. Beyond the heart, research data also support its renoprotective effects, such as reducing proteinuria and maintaining glomerular filtration rate, thereby improving outcomes in patients with renal failure. 4. These clinical protective effects have been further explored and confirmed by basic research, showing that Canagliflozin can alleviate renal fibrosis, improve tubular injury, and reduce glomerular damage under hyperglycemic conditions 5. By mitigating inflammatory responses and facilitating metabolic reprogramming, it exerts beneficial effects on the cardio-renal syndrome 6. By mitigating inflammatory responses and facilitating metabolic reprogramming, it exerts beneficial effects on the cardio-renal syndrome.

Mitochondrial dysfunction is increasingly recognized as a central player in the pathophysiology of AKI. Mitochondria, as the energy powerhouse of cells, are critical for tubular cell survival and function. Insults such as ischemia-reperfusion or sepsis disrupt mitochondrial homeostasis, leading to ATP depletion, oxidative stress, and activation of apoptotic pathways, which collectively exacerbate tubular damage. Mitochondrial biogenesis, a process that replenishes the mitochondrial pool, has emerged as a key defense mechanism against these injuries. Recent studies suggest that enhancing mitochondrial biogenesis could alleviate AKI by restoring energy balance and reducing oxidative damage, making it a promising therapeutic target.

Recent findings highlight a complex interplay among AMPK, LPS-related kidney damage, and SGLT2 inhibitors (SGLT2i), offering insights into their potential therapeutic connections. AMPK, a key metabolic regulator, mitigates LPS-induced kidney damage by reducing inflammation, oxidative stress, and fibrosis while promoting autophagy and energy homeostasis 7, 8. LPS, a major driver of sepsis-related acute kidney injury, activates TLR4 signaling, leading to systemic inflammation, oxidative damage, and microvascular dysfunction, which exacerbate renal injury 9, 10. SGLT2 inhibitors, traditionally used for glucose control, have demonstrated renoprotective effects by lowering intraglomerular pressure, enhancing mitochondrial function, and attenuating inflammation and oxidative stress. Notably, recent studies suggest that SGLT2i may indirectly activate AMPK or share overlapping protective pathways, such as reducing inflammation and metabolic stress 11-13. This emerging interplay indicates that targeting AMPK activation and leveraging the anti-inflammatory and metabolic effects of SGLT2i could synergistically counteract LPS-induced renal damage, providing a promising strategy for managing sepsis-associated kidney dysfunction.

Building on this foundation, using lipopolysaccharide (LPS)-induced septic AKI in mice and human proximal tubular cells (HK-2), we investigated the effects of canagliflozin on mitochondrial homeostasis, tubular cell survival, and renal function. We further evaluated the dependency of these effects on AMPKα1 by employing genetic knockout models and pharmacological inhibitors.

Here, we demonstrate that canagliflozin significantly alleviates LPS-induced renal dysfunction by restoring mitochondrial biogenesis and reducing oxidative stress, inflammation, and apoptosis in tubular cells. These outcomes are facilitated via the stimulation of the AMPKα1/PGC1α/NRF1 pathway, as AMPKα1 inhibition abolished the mitochondrial protective effects of canagliflozin. Our findings not only uncover a novel mechanism underlying canagliflozin's renoprotective effects but also establish the AMPKα1 as as an essential treatment focus for alleviating septic AKI.

Our study addresses a critical gap in AKI research by linking the pharmacological benefits of canagliflozin to mitochondrial biogenesis and AMPKα1 activation, providing a molecular basis for its therapeutic potential in septic AKI. By integrating cellular, molecular, and functional analyses, our work lays the groundwork for future clinical investigations aimed at translating these findings into effective therapies for AKI 14, 15.

## Materials And Methods

### Ethics approval

All animal experiments were approved by the Nanfang Hospital, Southern Medical University (NO. GSE148702). Animals were handled following the US National Institutes of Health Guide for the Care and Use of Laboratory Animals.

### Mice

WSeptic AKI was induced by administering 12 mg/kg lipopolysaccharide via injection, following a previously established protocol. Mice were assessed 24 hours post-treatment. In an independent study, mice received a daily dose of 10 mg/kg canagliflozin for a week prior to the onset of septic acute kidney injury 16, 17. Furthermore, to suppress AMPKα1 function, animals were pretreated with compound C (10 mg/kg) 3 hours prior to canagliflozin administration, and this dosing schedule was maintained for a week preceding the onset of septic acute kidney injury 18-20.

### Immunofluorescence

A standardized immunofluorescence staining procedure was used to prepare samples for microscopy, enabling the detection and localization of target proteins and structures 21-23.

### ELISA

The levels of Scr and BUN were quantified using ELISA kits specifically designed for mouse samples. To evaluate the degree of programmed cell death, caspase-3 activity was quantified employing a commercially available immunoassay 24, 25. This kit provided a sensitive and quantitative means of detecting caspase-3 activity, which is a key indicator of apoptotic cell death 26, 27. The concentrations of various antioxidant enzyme were determined in the culture media of HK-2 cells using ELISA kits. Nonetheless, these ELISA kits were used according to the manufacturers' protocols to ensure accurate and reliable results 28-32.

### Evaluation of mitochondrial performance and oxidative stress visualization

The mitochondrial potential wasevaluated with a fluorescent probe, following a previously established protocol. To evaluate the levels of ROS within the mitochondria and the cell as a whole, two specialized kits were employed. The MitoSOXTM Red Mitochondrial ROS Kit (#M36008, Invitrogen) was used to specifically measure mitochondrial ROS, while the Image-ITTM LIVE Green ROS Kit (#I36007, Invitrogen) was utilized to detect cellular ROS 33. These kits provided a sensitive and quantitative means of assessing ROS levels, allowing for a comprehensive understanding of oxidative stress within the cell. The production of adenosine triphosphate (ATP), a key indicator of cellular energy metabolism, was determined. This kit enabled the accurate measurement of ATP levels 27, 34, 35. By using these specialized kits, a detailed understanding of mitochondrial function, ROS levels, and cellular energy metabolism could be obtained, facilitating the investigation of complex cellular processes and the evaluation of potential therapeutic strategies 30, 36, 37.

### Evaluation of cell viability

The HK-2 cell line was maintained in a controlled environment and treated with LPS to induce septic AKI-like conditions 38, 39. Canagliflozin was added to the cells to investigate its potential protective effects, and AMPKα1 activity was inhibited using compound C to examine its involvement in canagliflozin's effects. Cell viability was measured using a CCK-8 assay, facilitating the assessment of canagliflozin's protective effects on HK-2 cells under septic AKI-like conditions 40, 41. This study utilized an *in vitro* model to elucidate the mechanisms of canagliflozin's potential protective effects and the contribution of AMPKα1 to these effects 42, 43.

### qRT-PCR analysis

The expression of target genes was assessed through a multi-step process involving RNA extraction, reverse transcription, and quantitative PCR analysis 44. The data were subsequently normalized to an internal control and subjected to a comparative quantification method to determine the fold changes in transcript levels, with a focus on genes involved in inflammatory responses 45, 46.

### TUNEL staining

Apoptotic cells in kidney tissue were identified using TUNEL staining, a reliable method that involves labeling fragmented DNA 47, 48.

### Western blot analysis

The analysis of protein expression involved a sequential process, comprising cell disruption, protein quantification, gel electrophoresis, and antibody-mediated detection 49. The resulting protein profiles were subsequently visualized using a chemiluminescent detection system and analyzed using a digital imaging platform, yielding a precise and reliable assessment of protein abundance 50, 51.

### Measurement of ATP and lactate

A multifaceted experimental strategy was devised to investigate the bioenergetic properties of HK-2 cells. Following a 24-hour cultivation period, the cells' energetic state was evaluated through the quantification of ATP content using a specialized assay, while the accumulation of lactate in the surrounding medium was also monitored 52. To ensure the accuracy and reliability of the results, the data were calibrated to account for any discrepancies in cellular density, thereby yielding a detailed and nuanced portrayal of the cells' metabolic landscape 53.

### Statistical analysis

IBM SPSS Statistics 25.0 was used for data analysis, with t-tests, ANOVA, and post hoc tests applied to determine significance at P < 0.05 54.

## Results

### Canagliflozin protects renal function in lipopolysaccharide kidney injury

**Figure 1:**
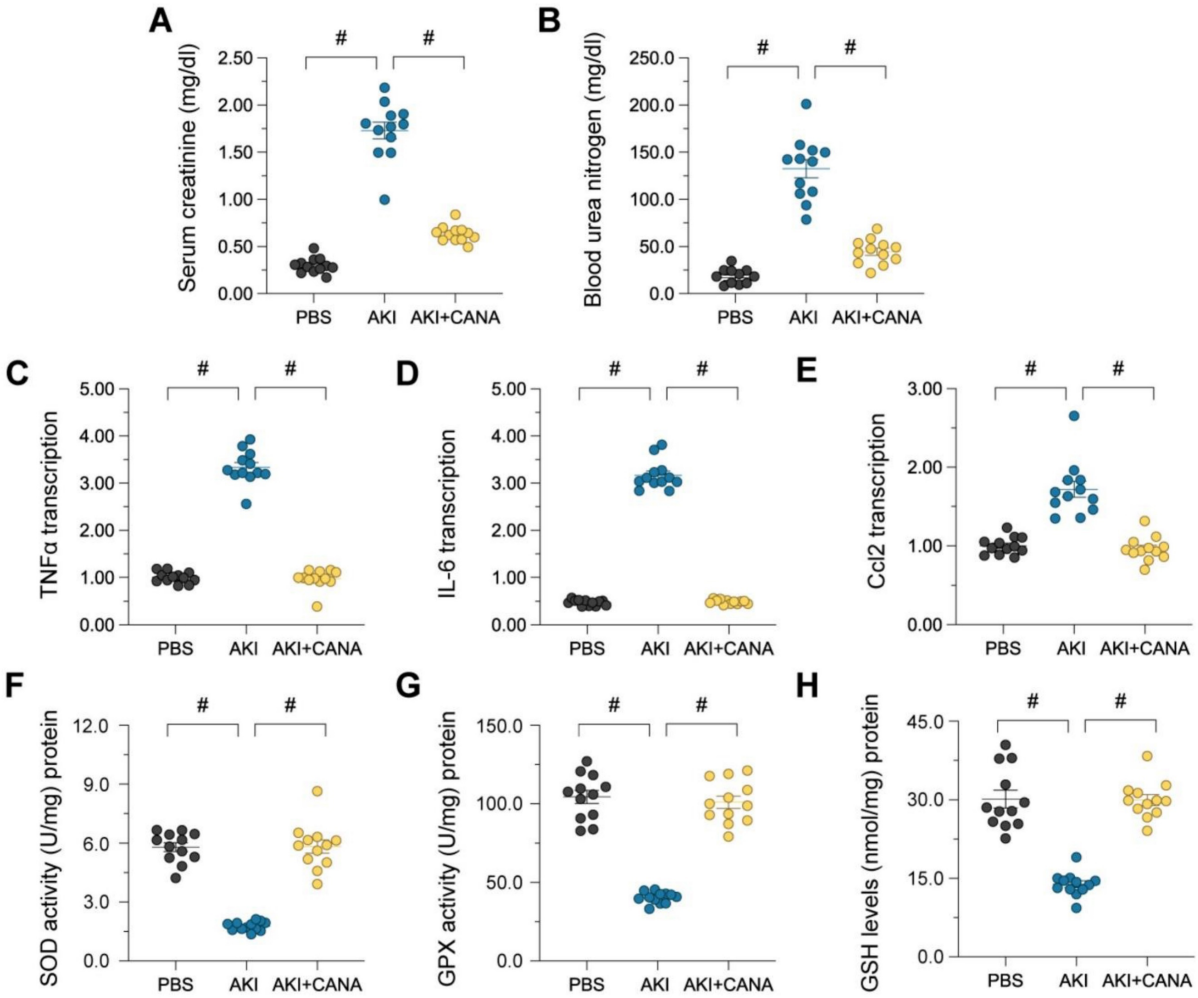
** Canagliflozin preserves kidney function in lipopolysaccharide-induced AKI.** WT mice were injected with lipopolysaccharide (12 mg/kg body weight) to induce septic AKI, and were evaluated after 24 hours. Canagliflozin (10 mg/kg/day) was administered to the mice for seven days prior to the induction of septic AKI. **(A, B)** ELISAs were used to determine BUN and Scr levels in mice with septic AKI. **(C-E)** RNA was isolated from the kidneys after septic AKI, and qRT-PCR was used to assess the transcription of *IL-6*, *Ccl2* and *TNFα*. **(F-H)** ELISAs were used to measure the activities of anti-oxidative enzymes such as GSH, SOD and GPX in kidney tissues. #p<0.05.

Administering canagliflozin for seven days before AKI induction enhanced kidney performance, demonstrated by decreased blood urea nitrogen (BUN) and serum creatinine (Scr) concentrations. (Figure 1A-B). LPS-triggered AKI additionally elevated pro-inflammatory cytokines, such as IL-6, Ccl2, and TNFα (Figure 1C-E), while Inhibiting antioxidant enzymes like GSH, SOD, and GPX (Figure 1F-H), resulting in oxidative stress and kidney damage. Canagliflozin pretreatment lowered cytokine expression and restored antioxidative defenses, demonstrating its protective effects through anti-inflammatory and antioxidative mechanisms.

### Canagliflozin protects against tubular cell death in AKI

**Figure 2:**
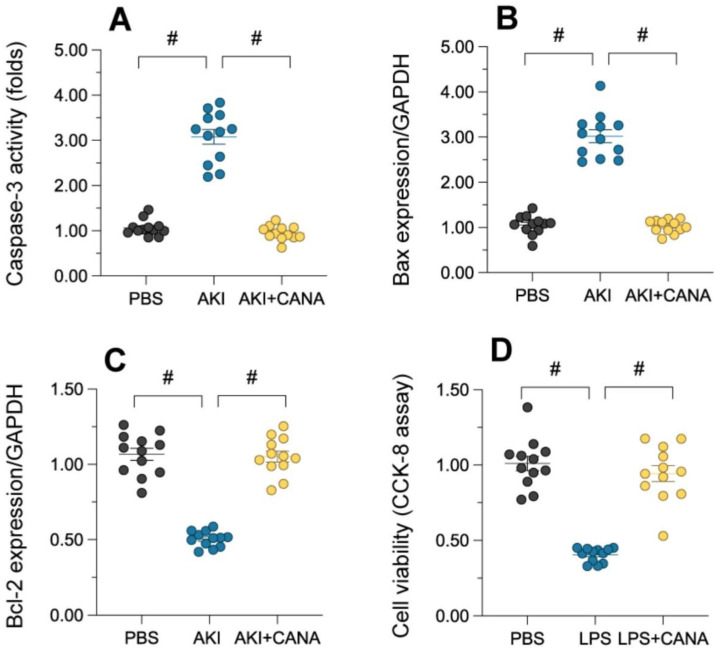
** Canagliflozin reduces tubular death during AKI.** WT mice were injected with lipopolysaccharide (12 mg/kg body weight) to induce septic AKI, and were evaluated after 24 hours. Canagliflozin (10 mg/kg/day) was administered to the mice for seven days prior to the induction of septic AKI. To establish an *in vitro* model of septic AKI in tubular cells, HK-2 cells were challenged with lipopolysaccharide (10 μg/mL) for 24 hours. Control cells were treated with PBS. Canagliflozin (10 μM) was added to the HK-2 cell culture medium 24 hours before lipopolysaccharide treatment. **(A)** ELISA was used to determine the activity of caspase-3. **(B-C)** Proteins were isolated from HK-2 cells, and Western blotting was used to assess the expression of Bax and Bcl-2. **(D)** A CCK-8 assay was used to analyze cell viability. #p<0.05.

Tubular cell death, a hallmark of acute kidney injury (AKI), was assessed by measuring caspase-3 activity in lipopolysaccharide (LPS)-treated kidneys. LPS significantly increased caspase-3 activity, indicating heightened apoptosis, while canagliflozin reduced it to near-normal levels (Figure 2A). Western blot analysis showed that LPS LPS increased the expression of pro-apoptotic Bax and decreased anti-apoptotic Bcl-2, disrupting the Bax/Bcl-2 balance and promoting cell death (Figure 2B-C). Canagliflozin restored this balance, enhancing cell survival. *In vitro* studies using HK-2 cells further confirmed these effects (Figure 2D). These results demonstrate that canagliflozin prevents tubular cell death in AKI by modulating apoptosis and promoting cell viability.

### Canagliflozin mitigates mitochondrial dysfunction in AKI

**Figure 3:**
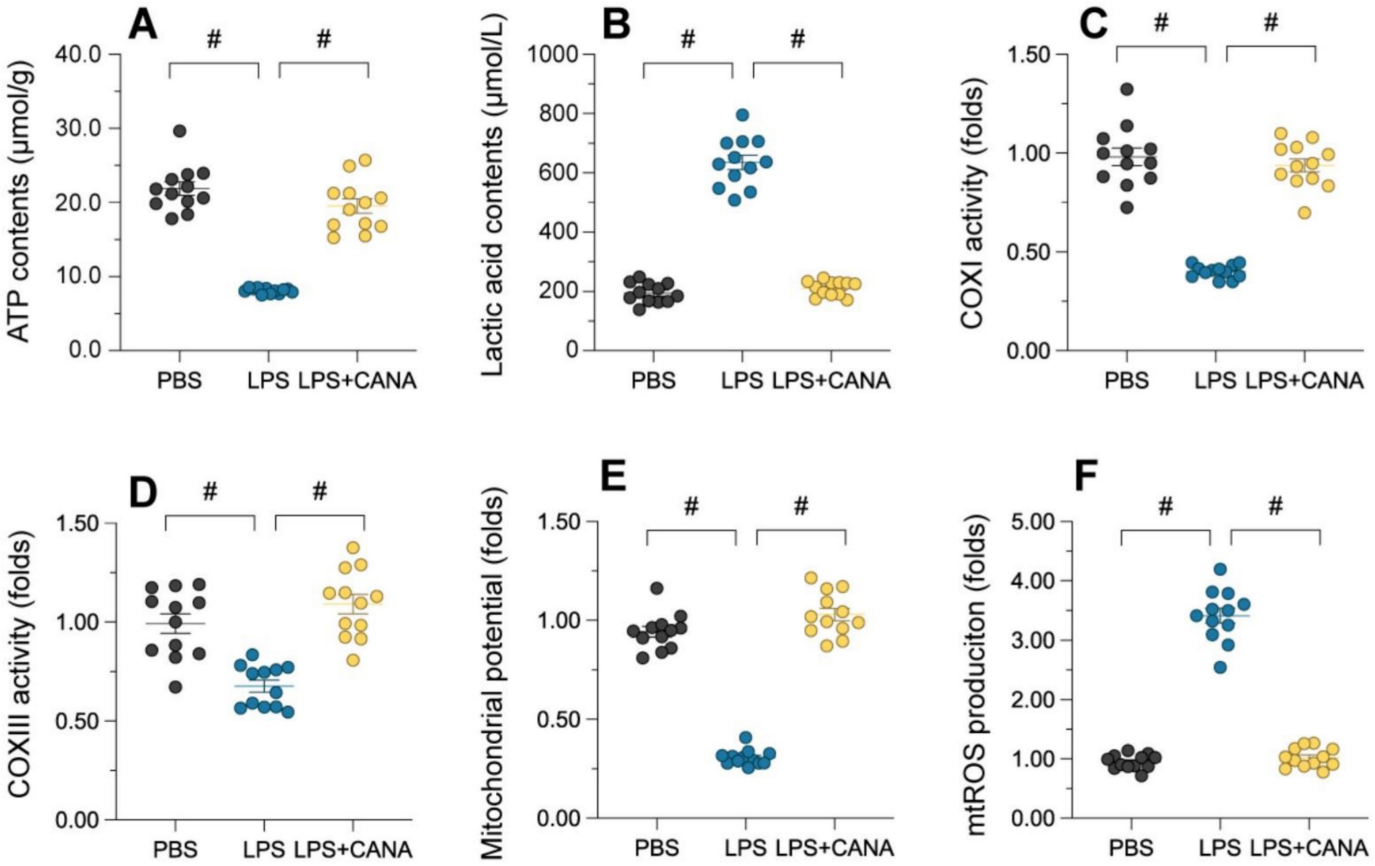
** Canagliflozin ameliorates AKI-induced mitochondrial dysfunction.** To establish an *in vitro* model of septic AKI in tubular cells, HK-2 cells were challenged with lipopolysaccharide (10 μg/mL) for 24 hours. Control cells were treated with PBS. Canagliflozin (10 μM) was added to the HK-2 cell culture medium 24 hours before lipopolysaccharide treatment. **(A, B)** ELISAs were used to measure ATP production and lactic acid levels in HK-2 cells treated with lipopolysaccharide. **(C, D)** ELISAs were used to analyze alterations in mitochondrial respiratory complex I and III activity. **(E)** A JC-1 probe was used to measure the mitochondrial membrane potential. **(F)** Immunofluorescence staining was used to display mitochondrial ROS accumulation in HK-2 cells. #p<0.05.

Maintaining mitochondrial integrity is essential for tubular cell survival, as mitochondrial damage often precedes cell death. To assess whether canagliflozin supports tubular cells by preserving mitochondrial function, we evaluated its impact on mitochondrial health in HK-2 cells. ATP production, a key mitochondrial activity, was significantly reduced in lipopolysaccharide (LPS)-treated cells, accompanied by elevated lactic acid levels (Figure 3A-B). Canagliflozin restored ATP synthesis and reduced lactic acid, suggesting improved mitochondrial function. Further analysis revealed that LPS suppressed the activity of complexes I/III, critical for ATP production, while canagliflozin reversed this inhibition (Figure 3C-D). Similarly, LPS disrupted mitochondrial membrane potential, as measured by JC-1 staining, whereas canagliflozin stabilized it, highlighting its protective role (Figure 3E). LPS-induced mitochondrial dysfunction also triggered an accumulation of mitochondrial reactive oxygen species (ROS), but canagliflozin effectively prevented this oxidative stress (Figure 3F). Collectively, these findings demonstrate that canagliflozin safeguards mitochondrial function and homeostasis in LPS-treated tubular cells, promoting their survival under stress.

### AMPK and mitochondria function as the downstream of canagliflozin

**Figure 4:**
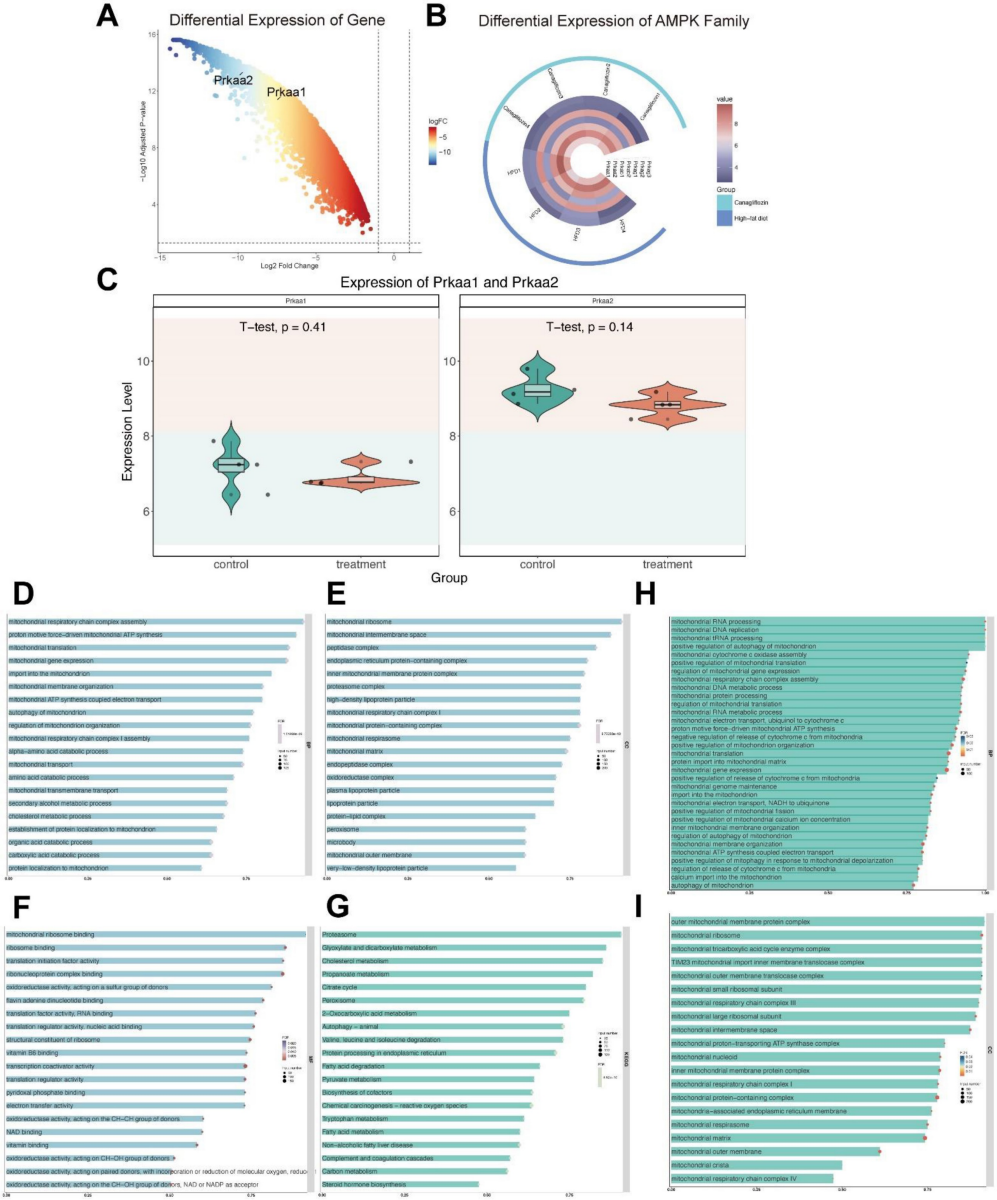
** AMPK And mitochondria funciton downstream of Canagliflozin. (A)** Volcano plot showing differential gene expression. The x-axis represents the log2 fold change in gene expression, while the y-axis indicates the -log10 adjusted p-value. Genes with greater negative log2 fold changes are significantly downregulated in the treatment group. The higher the position of a point, the stronger its statistical significance, and the farther from zero, the larger the expression change. **(B)** Circular heatmap displaying the expression patterns of seven AMPK subunit genes (*Prkaa1, Prkaa2, Prkab1, Prkab2, Prkag1, Prkag2, Prkag3*) in the canagliflozin-treated group (red, *n*=4) and HFD group (blue, *n*=4). Each ring represents a sample, with color intensity reflecting gene expression levels (range: 4-8). **(C)** Violin plots illustrating the expression distributions of *Prkaa1* and *Prkaa2* between the control and treatment groups. The y-axis represents gene expression levels, while the violin shape indicates probability density. Internal boxplots denote the median and interquartile range. Statistical analysis by t-test showed no significant differences for *Prkaa1* (p=0.41) or *Prkaa2* (p=0.14).** (D)** Bar chart showing the top 20 enriched biological processes (BPs), primarily related to mitochondrial function, energy metabolism, and amino acid metabolism. The x-axis represents the enrichment ratio, and the y-axis lists biological processes. Bar color intensity indicates FDR significance, length reflects the enrichment ratio, and width represents the number of genes involved. **(E)** Bar chart of the top 20 enriched cellular components (CCs), predominantly mitochondrial structures, protein complexes, and lipoprotein particles. Key components include mitochondrial ribosomes, intermembrane spaces, inner membrane protein complexes, and various lipoprotein particles. **(F)** Top 20 enriched molecular functions (MFs), focusing on translation regulation, oxidoreductase activity, and cofactor binding. Functions such as ribosome binding, initiation factor activity, and binding of cofactors (e.g., FAD, NAD, and vitamin B6) are significantly enriched. (G) Bar chart of the top 20 enriched KEGG pathways, including energy metabolism, lipid metabolism, and amino acid metabolism. Significant pathways include the TCA cycle, cholesterol metabolism, proteasome, and branched-chain amino acid degradation. Important pathways such as peroxisome, autophagy, and endoplasmic reticulum protein processing are also enriched. **(H)** Enriched mitochondrial biological processes. The x-axis shows the enrichment ratio, and the y-axis lists specific processes. Bar color intensity reflects FDR significance, bar length represents the enrichment ratio, and bar width indicates the number of genes involved. Key processes include mitochondrial RNA processing, DNA replication, translation regulation, electron transport chain assembly, ATP synthesis, cytochrome c release, mitochondrial autophagy, and calcium ion transport, emphasizing the role of mitochondria in energy metabolism and homeostasis. **(I)** Enriched mitochondrial cellular components. The x-axis represents the enrichment ratio, and the y-axis lists components. Bar color intensity denotes FDR significance, length reflects the enrichment ratio, and width indicates the number of genes involved. Significantly enriched structures include mitochondrial ribosomes (small and large subunits), outer membrane complexes, the TIM23 translocase complex, respiratory chain complexes (I/III/IV), the ATP synthase complex, cristae, and mitochondria-associated ER membranes, reflecting the diverse functional regions of mitochondria.

Our investigation into the downstream effects of canagliflozin involved the analysis of a publicly available dataset, GSE148702, which comprised data from diet-induced obesity (DIO) C57BL/6J mice treated with canagliflozin. These mice were compared to both high-fat diet (HFD) controls and weight-matched controls subjected to caloric restriction. Through differential gene expression analysis utilizing the limma package, we identified a significant upregulation of genes encoding AMPK subunits, specifically Prkaa1 and Prkaa2 (Figure 4A). The expression profiles of the AMPK gene family were visualized through heatmaps (Figure 4B), while violin plots illustrated the expression distribution of Prkaa1 and Prkaa2 between the canagliflozin-treated and HFD groups (Figure 4C).

To further elucidate the functional implications of these findings, we conducted a GO analysis. Within the molecular function (MF) category, terms such as vitamin B6 binding, pyridoxal phosphate binding, and oxidoreductase activity were highly enriched. The cellular component (CC) analysis predominantly highlighted enrichment in mitochondrial protein-containing complexes, mitochondrial matrix, and inner mitochondrial membrane protein complexes (Figure 4D-G).

KEGG analysis showed enrichment in pathways associated with peroxisome, and cholesterol metabolism. By analyzing enriched BP and CC categories directly associated with mitochondrial processes, we were able to provide a focused visualization of mitochondrial-related pathways. These results collectively underscore the pivotal role of differentially expressed genes (DEGs) in modulating mitochondrial function (Figure 4D-G).

A more in-depth analysis of mitochondrial-enriched biological processes (Figure 4H) was conducted to gain deeper insights into how canagliflozin influences mitochondrial biogenesis and functionality. The bar chart illustrates the most significantly enriched mitochondrial-related biological processes. The intensity of bar colors denotes the significance level (FDR), the bar length reflects the enrichment ratio, and the bar width indicates the number of genes involved in each process. Notable enriched processes included mitochondrial RNA processing, mitochondrial DNA replication, mitochondrial translation regulation, electron transport chain assembly, ATP synthesis, cytochrome c release regulation, mitochondrial autophagy, and calcium ion transport. These findings emphasize the critical function of mitochondria in energy generation, metabolic control, and cellular equilibrium.

Similarly, the bar chart in Figure 4I illustrates the most significantly enriched mitochondrial cellular components. The x-axis represents the enrichment ratio, while the y-axis lists specific cellular components. The color intensity of the bars reflects the FDR significance, bar length denotes the enrichment ratio, and bar width represents the number of genes associated with each component. The analysis highlights the significant enrichment of various mitochondrial structures, including the outer membrane protein complex, mitochondrial ribosome, tricarboxylic acid cycle enzyme complex, TIM23 translocase complex, respiratory chain complexes, ATP synthase complex, ribosomes, mitochondrial matrix, cristae, and mitochondria-associated endoplasmic reticulum membranes.

Together, these enriched processes and structural components underscore the central role of mitochondrial regulatory mechanisms in maintaining cellular energy metabolism and homeostasis. The data strongly suggest that canagliflozin exerts its therapeutic effects, at least in part, through the modulation of key mitochondrial functions, thereby highlighting its potential in managing obesity-related metabolic dysfunctions.

### Canagliflozin promotes mitochondrial generation through the AMPKα1/PGC1α/NRF1 axis

**Figure 5:**
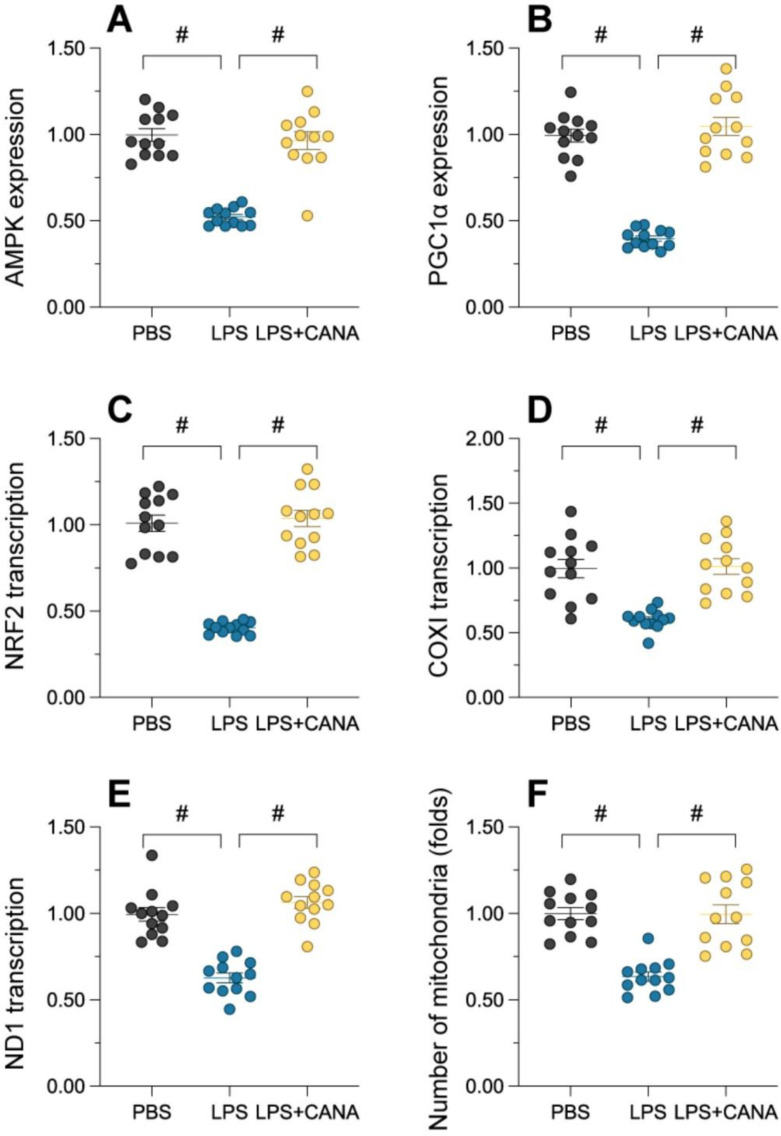
** Canagliflozin restores mitochondrial biogenesis through the AMPKα1/PGC1α/NRF1 pathway.** To establish an *in vitro* model of septic AKI in tubular cells, HK-2 cells were challenged with lipopolysaccharide (10 μg/mL) for 24 hours. Control cells were treated with PBS. Canagliflozin (10 μM) was added to the HK-2 cell culture medium 24 hours before lipopolysaccharide treatment. **(A-B)** Expression of AMPKα1 and PGC1α levels in HK-2 cells. **(C-E)** qRT-PCR was used to analyze the transcription of *AMPKα1*, *NRF1*, *ND1* and *COX1* in HK-2 cells treated with lipopolysaccharide. **(F)** Immunofluorescence staining was used to display the number of mitochondria in HK-2 cells. #p<0.05.

Generation of mitochondria is essential for replenishing damaged mitochondria and mitigating dysfunction. To evaluate whether canagliflozin's mitochondrial protection involves biogenesis, we examined key regulators of this process. Immunofluorescence showed that lipopolysaccharide (LPS) significantly suppressed AMPKα1 and PGC1α abundance, while canagliflozin restored their levels (Figure 5A-B). Similarly, NRF1 was negatively controlled by LPS and reversed through canagliflozin (Figure 5C), indicating activation of this axis. Lipopolysaccharide exposure disrupted the expression of key mitochondrial transcripts, including those encoding the alpha subunit of the mitochondrial respiratory complex I and the core subunit of the mitochondrial cytochrome c oxidase, reducing mitochondrial mass and viability in HK-2 cells (Figure 5D-F). Canagliflozin reversed these effects, restoring ND1 and COX1 expression and increasing mitochondrial population and health. Overall, canagliflozin enhances mitochondrial gene transcription and increases functional mitochondria, reducing damage caused by LPS. This highlights the canagliflozin-induced activation of the AMPKα1/PGC1α/NRF1 cascade is a crucial determinant of its mitochondrial-protective effects, underscoring the importance of this signaling axis in maintaining mitochondrial integrity and function.

### AMPKα1 Inhibition blocks canagliflozin's mitochondrial protective effects

**Figure 6:**
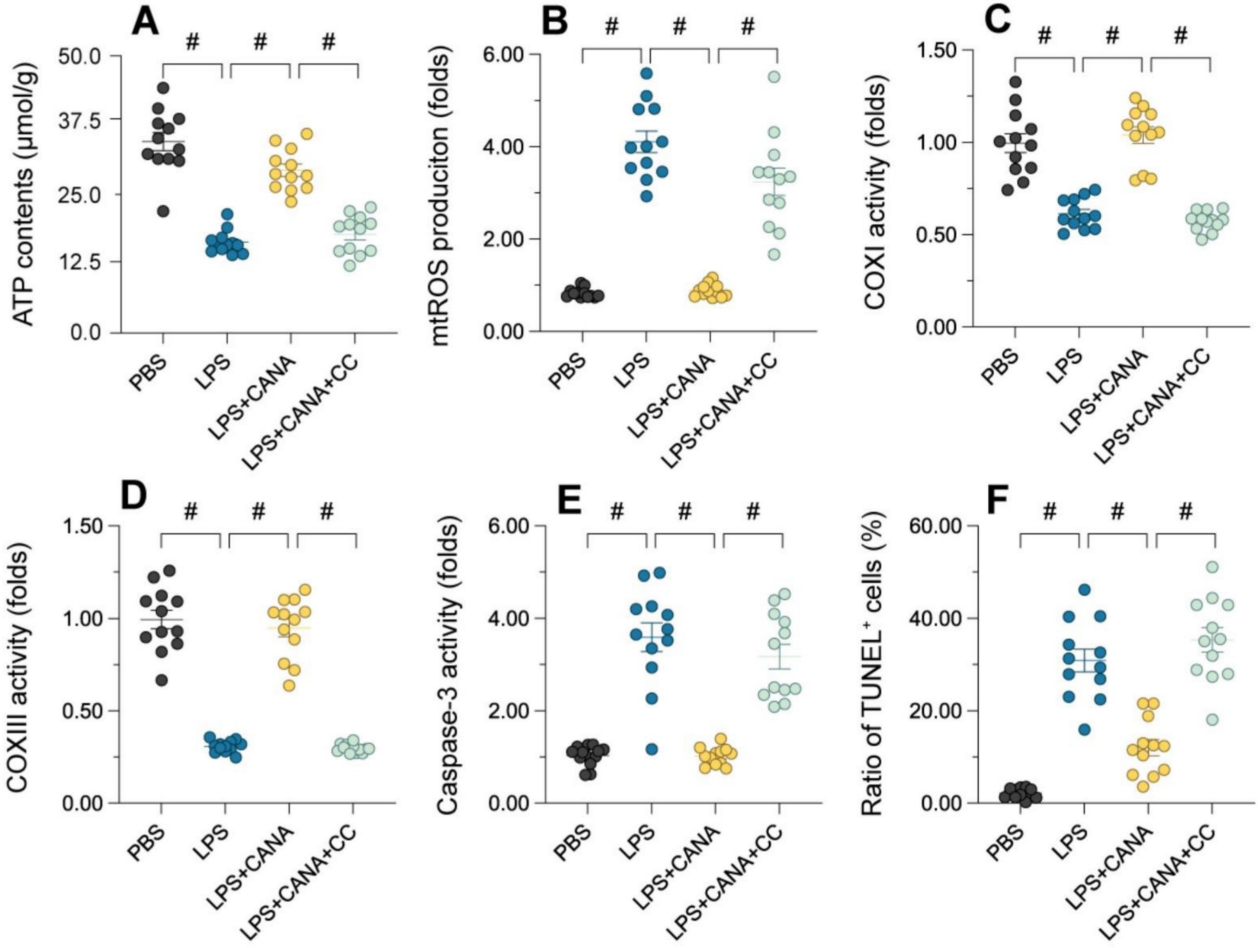
** Inhibition of AMPKα1 abolishes the mitochondrial protective effects of canagliflozin in tubular cells.** To establish an *in vitro* model of septic AKI in tubular cells, HK-2 cells were challenged with lipopolysaccharide (10 μg/mL) for 24 hours. Control cells were treated with PBS. Canagliflozin (10 μM) was added to the HK-2 cell culture medium 24 hours before lipopolysaccharide treatment. To inhibit the activity of AMPKα1, HK-2 cells were treated with CC (10 mg/kg) three hours before canagliflozin treatment. **(A)** ELISAs were used to measure ATP production in HK-2 cells treated with lipopolysaccharide. **(B)** Immunofluorescence staining was used to detect mitochondrial ROS accumulation in HK-2 cells. **(C-E)** An ELISA was used to analyze the activity of caspase-3 and mitochondrial respiration complex I/III. **(F)** TUNEL staining was used to determine the number of dead HK-2 cells upon lipopolysaccharide treatment. #p<0.05.

To validate the involvement of the AMPKα1/PGC1α/NRF1 pathway, we used compound C (CC), a selective AMPKα1 antagonist, to assess its impact on mitochondrial function. Pre-treatment with CC in HK-2 cells abolished the beneficial effects of canagliflozin. While canagliflozin elevated ATP levels in LPS-treated cells, CC pre-treatment negated this improvement (Figure 6A). Similarly, canagliflozin reduced mitochondrial ROS accumulation induced by LPS, but CC reversed this effect, showing that AMPKα1 is essential for its antioxidant action (Figure 6B). Canagliflozin also restored mitochondrial respiratory complex activity (Figure 6C-D) and prevented LPS-induced caspase-3 activation (Figure 6E), both of which were disrupted by CC. TUNEL staining further confirmed that canagliflozin reduced apoptosis in tubular cells, an effect nullified by CC treatment (Figure 6F). These results demonstrate that AMPKα1 inhibition prevents canagliflozin from maintaining mitochondrial integrity and reducing cell death under LPS-induced stress. In summary, the mitochondrial and cellular protective effects of canagliflozin depend on AMPKα1 activation. Blocking AMPKα1 with CC disrupts mitochondrial homeostasis, highlighting the pivotal role of the pathway in mitigating LPS-induced damage and promoting tubular cell survival.

## Discussion

Our study builds on the growing body of research highlighting the therapeutic potential of SGLT2i, particularly canagliflozin, in renal and cardiovascular protection. Unlike earlier investigations focusing on diabetic nephropathy or cardiorenal syndrome, we explored the effects of canagliflozin in septic AKI, a condition with complex pathophysiology involving inflammation, oxidative stress, and mitochondrial dysfunction 55, 56. Notably, previous studies demonstrated that canagliflozin alleviates renal oxidative stress and fibrosis in diabetic patients via anti-inflammatory and antioxidative mechanisms ings extend this knowledge by revealing a novel pathway: canagliflozin protects renal tubular cells in septic AKI through the AMPKα1/PGC1α/NRF1 axis, which restores mitochondrial regeneration and homeostasis.

In contrast to studies emphasizing systemic metabolic improvements, such as reductions in glucolipotoxicity, we provnce at the molecular level, showing that canagliflozin maintains ATP production, stabilizes mitochondrial potential, and inhibits ROS. This is particularly significant because mitochondrial dysfunction has been implicated as a pivotal driver of tubular cell death in AKI 57, 58. Additionally, odependency of canagliflozin's protective effects on AMPKα1 activation, which has been identified in other contexts, such as ischemia-reperfusion injury and cisplatin-induced nephrotoxicity 59-61. Our study is the first to confirm thiy in the context of septic AKI.

A key novelty of our study lies in the demonstration that canagliflozin's mitochondrial protective effects can be abolished by AMPKα1 inhibition 62-64. The use of pharmacological inhibitor compound C conclusively demonstrated that the pathway is indispensable for mediating canagliflozin's benefits. Additionally, we showed that canagliflozin reverses the suppression of complexes I/III caused by lipopolysaccharide (LPS), restoring mitochondrial function and promoting tubular cell survival 65, 66. This contrasts with prior studies that largely focused on canagliflozin's systemic anti-inflammatory properties, without delving into the specific molecular pathways driving mitochondrial recovery.

While our findings prov, several limitations warrant discussion. First, our study relied heavily on animal models and *in vitro* systems 67-69. Although these models closely mimic the pathophysiology of septic AKI, they cannot fully replicate the complexity of human disease. Future clinical study is necessary to confirm the translatability of these findings to patients with septic AKI 70-72. Second, while we focused on the AMPKα1/PGC1α/NRF1 axis, other pathways may also contribute to canagliflozin's protective effects 73-75. For instance, SGLT2i-mediated benefits have been linked to improved endothelial function and enhanced autophagy. Investigating the interplay between these mechanismside a more comprehensinding of canagliflozin's mode of action 76-78. Third, the study did not explore potential off-target effects of canagliflozin or compound C. Although the dependency on AMPKα1 was rigorously established, future studies should examine whether other cellular components, such as AMPKα2 or alternative PGC1α regulators, play supplementary roles in mediating these effects 79-82.

Several avenues for future research emerge from our findings. First, Investigations in a clinical setting are warranted to assess the potential risks and benefits of canagliflozin treatment in individuals suffering from septic acute kidney injury. This could include randomized controlled trials comparing canagliflozin with standard AKI treatments, such as supportive care and hemodialysis. Second, the mechanisms underlying AMPKα1 activation by canagliflozin require further exploration. Although our study implicates AMPKα1 as a critical mediator, the upstream signals that link canagliflozin to AMPKα1 activation remain unclear. Investigating these upstream regulators could reveal novel therapeutic targets for AKI. Third, given the multifaceted nature of AKI, it is essential to examine how canagliflozin interacts with other potential therapies. For instance, combining canagliflozin with NRF2 activators or autophagy enhancers could amplify its mitochondrial protective effects 83-88. Fourth, future studievestigate the long-terf canagliflozin on kidney function and structure post-AKI. Understanding whether canagliflozin prevents chronic kidney disease progression following septic AKI would provide valuable insights into its therapeutic potential. Finally, our study raises the intriguing possibility that other SGLT2 inhibitors may similarly activate the AMPKα1/PGC1α/NRF1 pathway. Comparative studies examining the efficacy of different SGLT2 inhibitors in septic AKI could help identify the most effective agent and optimize treatment strategies.

## Conclusion

Our study highlights the therapeutic potential of canagliflozin in septic AKI, emphasizing its role in preserving mitochondrial function and promoting tubular cell survival through the AMPKα1/PGC1α/NRF1 axis. By addressing the limitations of the current study and exploring the proposed future directions, we can further elucidate the role of SGLT2 inhibitors in renal protection and develop more effective treatments for AKI.
